# Peritonsillar Abscess Simulator: A Low-Cost, High-Fidelity Trainer

**DOI:** 10.21980/J85M0B

**Published:** 2022-04-15

**Authors:** Chad R Keller, Ivanna Nebor, David Choi, Kattia Moreno, Yash J Patil

**Affiliations:** * University of Cincinnati College of Medicine, Department of Otolaryngology Head and Neck Surgery, Cincinnati, OH

## Abstract

**Audience:**

Our reusable low-cost peritonsillar abscess simulator (PTA) simulator is designed to train emergency medicine (EM) residents, fellows, and medical students. Trainees who are interested in otolaryngology (OTL) or this specific disease may also benefit from this simulator.

**Introduction:**

Peritonsillar abscess is one of the most common deep infections 1 of the head and neck, accounting for 7589 consultations and 11069 hospital bed days in the UK between 2009–2010.^1^,^2^ Emergency medicine physicians commonly treat this pathology with surgical and medical modalities. Not only is this a common diagnosis, but there is a significant cost associated with the evaluation and management of primary PTA. 3

Demands for high-volume patient care and good patient outcomes are increasing in a medical climate of limited financial resources and resident work hours. Given these complexities, medical education is viewing simulation training, with proven success in various surgical specialties, as a valuable addition to resident education and patient safety. 3–5

The PTA is the collection of pus in the space between the palatine tonsil and its capsule. Successfully locating the abscess is crucial because it prevents the unwanted damage of nearby vascular structures, patient discomfort, and failure to treat the infection. Management of peritonsillar abscess is primarily surgical and includes incision and drainage (I & D), needle aspiration, or Quinsy tonsillectomy.

The simulator provides a realistic characteristic of typical PTA presentations, such as uvula deviation, swelling, trismus, and purulence during aspiration. While learning to drain a PTA, trainees must locate the infection with a needle without injury to the surrounding structures of the oral cavity and deep structures of the neck. The discomfort caused during this procedure can be unsettling for both physician and patient. Simulation use and testing enable the trainee to develop familiarity with handling instruments, increase comfort with the sequence of a procedure, and improve confidence in the ability to perform a procedure safely.^6^,^7^,^8^ Simulators provide improved patient outcomes and increased EM residents' comfort level.

**Educational Objectives:**

By the end of this training session, learners will be able to: 1) locate the abscess, 2) perform needle aspiration, and 3) develop dexterity in maneuvering instruments in the small three-dimensional confines of the oral cavity without causing injury to local structures.

**Educational Methods:**

Our PTA simulator was fabricated with a low-cost, non-degradable material and is the first known PTA simulator that used a validated survey for fidelity assessment. The simulator was fabricated using a silicone mold to mimic the oral cavity and oropharynx. A simulated abscess pocket consisting of saline encased in balloon material was placed in the proper anatomic location, allowing for abscess simulation on either side of the oropharynx model. The time to fabricate the model averaged 20 hours. The simulator was manufactured with low-cost materials at an expense of 45 USD and could be easily reproduced by any EM residency program.

**Research Methods:**

Twenty-one participants were instructed to expose and drain the simulated abscess. The model was evaluated using The Michigan Standard Simulation Experience Scale (MiSSES).^7^ Participants scored the simulator in five categories: Self-efficacy, fidelity, educational value, teaching quality, and the overall rating on a 5-point Likert scale of simulator. Overall rating and global evaluation scores were compared by groups (Group 1, Group 2) between training level (residents and attendings), specialty (emergency and otolaryngology), and previous experience (<5 or ≥5 drainages).

Convenience sampling was used to determinate the sample. Variables were summarized using the mean and standard deviation for continuous variables and percentages and frequencies for categorical variables. The MiSSES was scored as previously described in the literature.^7^ The Kolmogorov-Smirnov test was used to test for normal distribution of the variables. T-test for independent samples was performed to determinate if there exists a difference between groups in perception of a PTA simulator. The statistical analyses were performed using SPSS version 20.0 Armonk, NY: IBM.

**Results:**

Twenty-one participants were enrolled in the study: residents (n=15) and attending (n=6) from OTL and EM departments. The simulator’s plasticity allowed multiple attempts of needle aspiration and drainage without degradation and received high ratings on teaching quality, fidelity, and educational value. This PTA simulator achieved high fidelity ratings in the standard simulator’s assessment survey for realism of environment, simulation of trismus, uvular deviation, and realism of the mucosal surfaces. On the MiSSES, the model received positive ratings (range 3.6 to 4.9). The highest rating was on teaching quality (4.9), fidelity (4.6), and educational value (4.5) (Table 1). We found that self-efficacy and teaching quality sections were rated higher for those who had less experience (≥5 PTA drainage), while fidelity was rated higher for OTL. The overall rating average was 4 and was higher of attendings, OTL, and those with less experience. All comparisons between groups were not statically significant (Table 2). About 76% of participants found that the simulator can be used in training with slight improvement or no improvement needed. (Table 3)

**Discussion:**

With favorable participant ratings and comments, we believe that this tool can offer high-fidelity simulation at a low cost. Widespread use may be possible, allowing training of EM residents in performing instrumentation of PTA in a controlled simulation environment. We have created a reusable low-cost PTA simulator that achieved a high score fidelity in a standard simulator’s assessment survey.

**Topics:**

Peritonsillar abscess, oropharynx, emergency medicine residency, otolaryngology residency training.

## USER GUIDE

**Table:** 

List of Resources:	
Abstract	1
User Guide	4

**Learner Audience:**

Medical Students, Interns, Junior Residents, Senior Residents, Fellows

**Time Required for Implementation:**

Learning session should take 30 minutes: 10 minutes for introducing the technique, 5 minutes for important anatomical structure identification, 10 minutes for a step-by-step procedure, 5 minutes for final review.

**Recommended Number of Learners per Instructor:**

1–2 instructors for 5–8 learners

**Topics:**

Peritonsillar abscess, oropharynx, emergency medicine residency, otolaryngology residency training.

**Objectives:**

By the end of this training session, learners will be able to

1. Locate the abscess.
2. Perform needle aspiration.
3. Develop dexterity in maneuvering instruments in the small three-dimensional confines of the oral cavity without causing injury to local structures (ie, tongue, uvula, palate).

### Linked objectives and methods

Emergency room physicians and otolaryngologists often treat PTA with needle aspiration. Instructors will present trainees with the clinical scenario as well as the instruments required to interface with the simulator. Trainees will examine the oral cavity and identify the site of the peritonsillar abscess (Objective 1).

After localizing the side of the peritonsillar abscess, learners will expose the area with the provided instrumentation and lighting and insert the 10-cc syringe with 18-gauge needle into the abscess cavity, withdrawing as they pass through the mucosal surface of the simulator. Once they encounter the fluid reservoir, they will complete aspiration of the abscess (Objective 2).

Another unpleasant part during aspiration is possibly damaging adjacent structures with the needle (ie, tongue, uvula, palate). Usually this happens because of difficulty in manipulating instruments in the oral cavity in the presence of trismus, since this significantly impairs visualization. Our PTA simulator mimics trismus, and trainees can learn to manipulate instruments within a simulated environment (Objective 3).

### Recommended pre-reading for instructor

- Spiekermann C, Roth J, Vogl T, Stenner M, Rudack C. Potential of the Novel PTA Score to Identify Patients with Peritonsillar Inflammation Profiting from Medical Treatment. *Dis Markers*. 2018 May 28;2018:2040746. PMID: 29997713; PMCID: PMC5994576. doi: 10.1155/2018/2040746
- Powell J, Wilson JA. An evidence-based review of peritonsillar abscess. *Clin Otolaryngol*. 2012;37: 136–145.
- Vieira F, Allen SM, Stocks RM, Thompson JW. Deep neck infection. *Otolaryngol Clin North Am*. 2008; 41:459–83.

### Learner responsible content (LRC)

- Fernandez M.W., Desai B.K. Incision and Drainage of Peritonsillar Abscess. In: Ganti L. (eds) *Atlas of Emergency Medicine Procedures*. Springer: New York, NY. 2016. https://doi.org/10.1007/978-1-4939-2507-0_59
- Davis M, Alvarez Al’ai. Trick: Peritonsillar abscess drainage 3.0 - All the steps with added variations. ENT, Tricks of the Trade. Aug 9, 2019. https://www.aliem.com/tricks-peritonsillar-abscess-drainage-all-steps-variations/
- Roberts J, Hedges J, editors. *Clinical procedures in emergency medicine*. 5th ed. Philadelphia: Saunders; 2009:1184–9.

### Implementation Methods

Proctors for the simulation should instruct trainees that they are evaluating a patient with a likely PTA. Proctors must provide a clinical scenario that leads the trainee to believe that PTA is the likely diagnosis. Also, the clinical scenario must lead the trainee to believe that the PTA is located on the same side as the abscess reservoir that is placed on the simulator.

Each abscess should be the same size and location for each participant. The plasticity of the materials permitted repeated uses without any degradation in quality between the first and last participant. Participants complete the task with instruments that included a headlight, 18-gauge needle, tongue depressors, and 10-cc syringe as follows:

- Step 1: Locate abscess. Inspected and evaluated simulator tissue for the location of abscess based on clinical cues (eg, uvular deviation, proptosis of tonsil and tonsillar pillar, and trismus) characteristic of patient scenario.
- Step 2: Aspiration and drainage. Maneuver instruments using lighting within the simulator environment to aspirate infection with an 18-gauge needle.
- Step 3: Complete task. Dispose of sharps in proper containers.

All participants should be able to locate and drain the simulated abscess based upon the clinical clues above.

### List of items required to replicate this innovation

1. Laerdal Airway simulator (Laerdal, Wappingers Falls, NY)
2. Smooth-On Body Double (Smooth-On, Inc., Macungie, PA)
3. Laerdal simulator
4. Smooth-On Mann Ease Release 205
5. Smooth-On Dragon Skin
6. Smooth-On Silc-Pig pigment
7. Smooth-On Thi-Vex
8. Polyvinyl chloride (PVC) pipe adapter
9. Smooth-On Flex Foam It III
10. Saline

### Approximate cost of items to create this innovation

Actual material costs were 32 USD for the negative and positive mold material, 11 USD for the simulation mount material, and 1 USD per abscess pocket created.

### Detailed methods to construct this innovation

The model was developed using a life-casting technique by fabricating positive and negative molds to represent the oral cavity and oropharynx.

**Figure 1:**
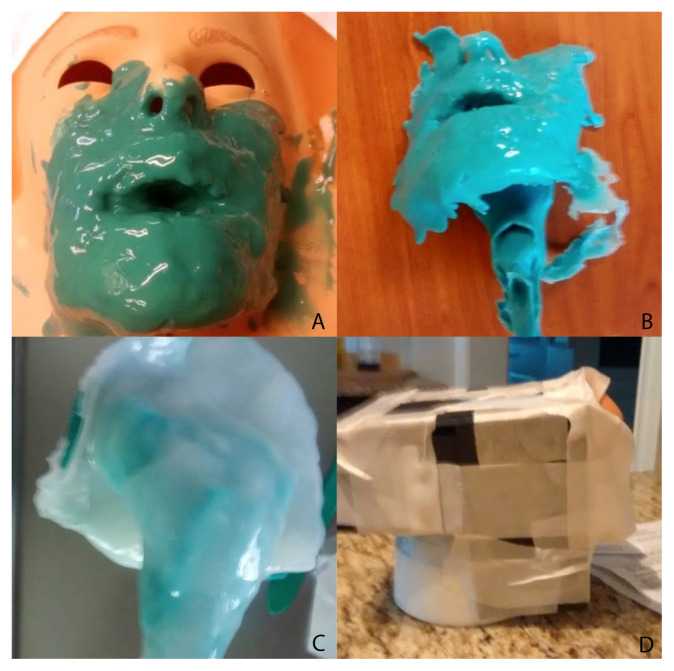
Fabrication of the peritonsillar abscess simulator. A, Casting of the negative mold. B, Negative mold before trimming. C, Casting of the positive mold. D, Scaffold used to make mounting unit.

**Figure 2:**
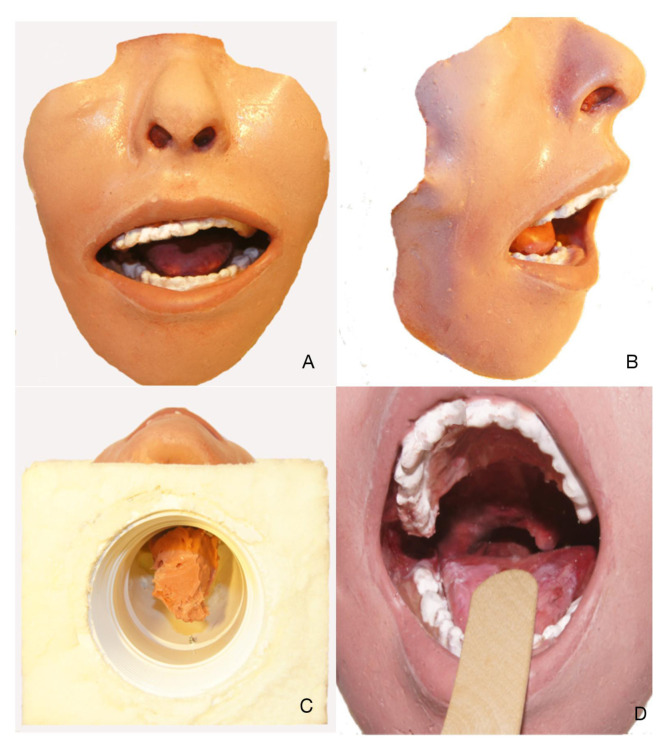
Different views of the peritonsillar abscess simulator. A, Front view. B, 45-degree view of simulator demonstrating 3-dimensional characteristics. C, Undersurface of simulator for placement of abscess pocket. D, Intraoral view as seen by the participant.

1. First, the negative mold was fabricated using a Laerdal Airway simulator (Laerdal, Wappingers Falls, NY). Smooth-On Body Double (Smooth-On, Inc., Macungie, PA) was poured into the simulator oropharynx and oral cavity and smoothed onto the exterior surface of the Laerdal simulator. This negative mold was subsequently trimmed and re-shaped to create a realistic oral cavity and oropharynx.
2. Smooth-On Mann Ease Release 205 was placed on the negative mold and brushed into the crevices; this step would prevent bonding of the silicone surfaces of the negative and positive molds and allow the two rubbers to easily separate after the casting process (Figure 1). The positive mold was created over the negative mold.
3. Smooth-On Dragon Skin, a clear and transparent material, was chosen as the positive mold material due to its ability to self-heal and allow for multiple attempts at needle aspiration and drainage. However, due to the translucent nature of the Smooth-On Dragon Skin, Smooth-On Silc-Pig pigment was added to the casting material to render an opacity to block ambient light and create a realistic coloring.
4. The pigmented mixture was then thickened with Smooth-On Thi-Vex to enable 360-degree casting, particularly in the dependent planes, to withstand gravitational forces. Additional Smooth-On Silc-Pig in a variety of hues was used in a moulage technique to replicate the simulated face, oral cavity, and oropharynx (Figure 2).
5. After making the positive mold, a 3-inch diameter, curved, polyvinyl chloride (PVC) pipe adapter was placed against the posterior surface of the oropharynx.
6. A cradle was constructed to hold the simulator using Smooth-On Flex Foam It III inside of a rectangular box constructed from foam core board around the PVC mount. The cradle and mount will serve as the port for placement of the abscess balloon on either side of the simulated oropharynx.
7. Once the foam had cured, the foam core board was removed, and the cradle and mount became a single unit. This allowed the simulator to be secure during instrumentation while preserving the ability to place the simulated abscess on either side of the oropharynx.
8. From the underneath side of the mount and cradle, a simulated abscess pocket consisting of saline encased in balloon material was placed in the proper anatomic location, allowing for abscess simulation on either side of the oropharynx model.

### Results and tips for successful implementation: Validated testing

Twenty-one participants completed the MiSSES immediately after using the simulator. 15 (71.4%) were male and 6 were female. Groups distribution is shown in Table 4.

Survey responses regarding the specific characteristics of the simulator along with the five categories ranged between a mean of 3.6 and 4.9 on the 5-point Likert scale. Respondents overall found all characteristics of the simulator positive, with a Likert score greater than 3 (Table 1). In the self-efficacy category, the simulator improves the confidence at performing instrumentation (3.8) and enhances the ability to instrument a PTA (3.8). In the fidelity category, realism of uvular deviation and the use of the simulator as a training tool received all 4.6 scores. In the educational category, the simulator as a useful tool to teach instrumentation received the highest of category of 4.5, while in the teaching quality category, instructors who were knowledgeable about PTA simulators gave the highest score (4.9). (Table 1). When analyzed by categories (self-efficacy, fidelity, educational value, teaching quality, and overall rating) between groups of training level, specialty, and experience, we found that self-efficacy and teaching quality sections were rated higher for those who had less experience (<5 PTA drainage), and fidelity and educational value were rated higher for OTL. The overall rating was higher for Attendings, OTL, and those with less experience. All group comparisons were not statistically significant. (Table 2)

When evaluating every question independently by groups, we found that attendings physicians rated significantly higher than residents on “the realism of simulation environment” (Question #6, p=0.018) and “the realism of simulation of trismus” (Question #7, p=0.009). Otolaryngologists rated significantly higher than EM practitioners on “the realism of uvular deviation” (Question #8, p=0.002) and “the realism of mucosal surfaces” (Question #10, p=0.029). The less experienced (< 5 PTA) rated higher on “helps to improve knowledge on PTA instrumentation” (Question #1, p=0.035).

In evaluating the readiness of the simulator for use as a training tool, participants checked one statement with which they most agree (1 = extensive improvements needed, 2 = minor improvement needed, 3 = should be improved slightly, 4 = no improvements needed). The simulator’s global responses were compared between attending and resident physicians, by medical specialty and by experience (≥5 PTA). Most of the participants, mostly residents (43%), EM (24%), and experienced (33.3%), agreed that “this simulator can be used in training but should be improved slightly” (52.3%) or no improvement needed (23.8%). No participant thought that extensive improvement was needed. (Table 3).

### Evaluating surgical simulator experiences

Previous studies have used the Objective Structured Assessment of Technical Skills (OSATS) for determining proficiency of a resident performing a specific task using surgical simulation.^5^,^9^,^10^ However, there are few standardized and subjective measures used for assessment of surgical simulator attributes.^11^ In developing a survey to evaluate surgical experiences with regard to surgical simulation validation, Seagull and Rooney^7^ sampled current literature to develop a standard subjective assessment tool for surgical simulation. Their tool, the Michigan Standard Simulation Experience Scale (MiSSES), assessed an entire range of domains. The questions in the MiSSES survey and in our study were categorized into self-efficacy, fidelity, educational value, teaching quality, overall rating and a global ranking. This is the first PTA simulator that is assessed using this standard simulation survey in an effort to compare realism and usefulness as a training tool by comparing groups of different abilities.^1^

### Discussion

Fidelity of simulation is typically correlated with cost. High-fidelity simulators are typically associated with higher costs. In our aim to develop and validate a PTA simulator with high fidelity and low cost, our simulator was fabricated with low-cost silicone materials and achieved acceptable fidelity, especially amongst experienced physicians. Of note, ratings were higher among physicians with more experience in head and neck anatomy and PTA instrumentation, and with those with post-graduate training. Those experienced with PTA appreciated the realistic appearance of the model’s anatomical details and nuances more than less experienced physicians, thus demonstrating this model’s face and content validity. For example, compared with EM physicians, seasoned Otolaryngology physicians rated uvular deviation simulation and the appearance of the mucosal surfaces as more realistic. Additionally, those with less experience felt that the simulation task helped to improve familiarity with PTA instrumentation. Lastly, attending physicians rated the simulation of environment and simulation of trismus as realistic. This low-cost PTA simulator was overall rated higher from attendings, OTL, and the less experienced, showing its fidelity and training usefulness. Moreover, in a global ranking, about 76% of participants found that the simulator can be used in training with slight improvement or no improvement needed (Table 3).

### Limitations

This model has been designed for needle aspiration and not for I &D. Other limitations include the lack of a carotid vascular system, which would simulate a misguided placement of the instrument too deeply. Another limitation was the abscess itself. Constructed from a latex balloon and saline, it was a thinner consistency than real abscess material. Both limitations could easily be overcome by adding a red-fluid-filled carotid system into the polyvinyl chloride cradle unit and replacing saline with a higher viscosity, self-sealing system. As we continue our simulation training with this model, we intend to add a carotid artery system, modify the abscess system, and construct a patient face and head to serve as the cradling system. However, in our study, participants felt that the simulator with minor improvements would provide value as a training tool for physicians.

## Figures and Tables

**Table 1:** Aggregate survey responses by statement rated on a 5-point Likert scale (n=21) (1) Strongly disagree; (2) Somewhat disagree; (3) Neutral; (4) somewhat agree; (5) Strongly agree.

(Questionnaire adapted from Seagull et al.^6^)	
Statement	Mean (SD) Range (0–5)
**SELF EFFICACY**	
1.The simulator helped improve my knowledge of PTA instrumentation.	3.6 (1.02)
2.The simulator helped improve my confidence at performing instrumentation of the oral cavity.	3.8 (0.93) *
3.The simulator helped improve my ability to place an instrument into a peritonsillar abscess.	3.8 (1.0) *
4.The simulator helped improve my ability to perform FNA on a peritonsillar abscess independently.	3.7 (1.02)
**FIDELITY**	
5.The simulator used has adequately realistic characteristics/features.	4.3 (0.72)
6.The simulation environment is adequately realistic.	4.0 (0.63)
7.This simulator provided adequate simulation of trismus (inability to fully open mouth).	4.1 (1.06)
8.Realism of uvular deviation away from the side of the abscess was demonstrated adequately in this simulator.	4.6 (0.6) *
9.The external face of the simulator appeared realistic.	4.4 (0.68)
10.The mucosal surfaces appeared realistic.	4.3 (0.91)
11.Traveling through simulated tissue had similar tactile feedback compared to a real patient.	4.1(0.74)
12.The simulator and simulation are good training tools for knowledge of the instrumentation of oral cavity.	4.6 (0.51) *
13.The simulator and simulation are good training tools for skills in needle aspiration of peritonsillar abscess.	4.6 (0.50) *
**EDUCATIONAL VALUE**	
14.The simulator and simulation were critical at addressing trismus.	4.0 (0.97)
15.The simulator and simulation were critical at addressing uvular deviation away from the side of the abscess.	4.2 (0.75)
16.This simulation addresses essential functions/steps to perform this procedure in real life.	4.0 (1.12)
17.Practicing on this simulator will help me to gain skills needed to better perform the procedure on a patient.	4.3 (0.72)
18.This simulator is a useful tool to teach instrumentation of PTA.	4.5 (0.60) *
**TEACHING QUALITY**	
19.Instructor(s) were knowledgeable about the PTA simulator.	4.9 (0.30) *
20.Instructor(s) were able to convey material in a way that was understandable to me.	4.8 (0.44)
21.The learning materials improved my understanding of PTA drainage.	3.9 (1.09)
22.The resources we used improved my understanding of PTA drainage.	4.0 (1.18)
**OVERALL RATING**	4.0 (0.44)

* Highest score of each category

**Table 2:** Results by categories and group of subjects.

Groups	Training Level Mean (SD)			Training Specialty Mean (SD)			Experience Mean (SD)		
Section	R n=15	A n=6	P value	EM n=13	OTL n=8	P value	<5 PTA n=10	≥5 PTA n=11	P value
**Self-Efficacy**	3.8(1.07)	3.5(0. 47)	0.52	3.8(0.45)	3.5(1.44)	0.42	4.12(0.13)	3.3(1.13)	0.54
**Fidelity**	4.2(0.47)	4.5(0.31)	0.10	4.1(0.48)	4.5(0.33)	0.09	4.2(0.53)	4.4(0.37)	0.32
**Educational Value**	4.2(0.56)	4.4(0.52)	0.56	4.14(0.54)	4.5(0.47)	0.10	4.2(0.44)	4.4(0.62)	0.34
**Teaching Quality**	4.3(0.71)	4.5(0.51)	0.52	4.5(0.54)	4.2(0.79)	0.27	4.6(0.51)	4.2(0.74)	0.23
**Overall Rating**	4.2(0.48)	4.3(0.29)	0.52	4.2(0.38)	4.3(0.54)	0.60	4.23(0.33)	4.2(0.16)	0.71

R: Residents

A: Attending

EM: Emergency department.

OTL: Otolaryngology department.

<5 PTA: Less than 5 peritonsillar abscess drainage.

≥5 PTA: 5 or more peritonsillar abscess drainage.

No group had a significant difference (p<0.05) per section or general mean score.

**Table 3:** Global Ranking-evaluation of Simulator training for drainage of peritonsillar abscess (PTA) on 4-point scale. (1) Extensive improvement needed; (2) requires minor adjustment; (3) should be improved slightly; (4) no improvements needed.

Needs		Extensive Improvement N (% of the group)	Minor Adjustments N (% of the group)	Improved Slightly N (% of the group)	No improvements N (% of the group)	Total (N)
Group						
Training level	R	0	3(14.3)	9(42.9)	3(14.3)	15
	A	0	2(9.5)	2(9.5)	2(9.5)	6
Training specialty	EM	0	4(19)	6(23.8)	3(14.3)	13
	OTL	0	1(4.8)	5(23.8)	2(9.5)	8
Experience	< 5 PTA	0	3(14.3)	4(19)	3(14.3)	10
	≥ 5 PTA	0 2	(9.5)	7(33.3)	2(9.5)	11
Total		0	5(23.8)	11(52.3)	5(23.8)	21

R: Internal/Resident

A: Fellow/Attending

EM: Emergency department.

OTL: Otolaryngology department.

<5 PTA: Less than 5 peritonsillar abscess drainage.

≥5 PTA: 5 or more peritonsillar abscess drainage.

**Table 4:** Group Distribution.

	G1; N (%)	G2; N (%)	Total
Training Level	R	A	
	15 (71.4%)	6 (28.6%) 21	
Specialty	EM	OTL	
	13 (61.9%)	8 (38.1%) 21	
Experience	< 5 PTA	≥5PTA	
	10 (47.6%)	11 (52.4%)	21

G1: Group 1

G2: Group 2

R: Internal/Resident

A: Fellow/Attending

EM: Emergency department.

OTL: Otolaryngology department.

<5 PTA: Less than 5 peritonsillar abscess drainage.

≥5 PTA: 5 or more peritonsillar abscess drainage.
